# Aqueous *Nyctanthes arbortristis* and doxorubicin conjugated gold nanoparticles synergistically induced mTOR-dependent autophagy-mediated ferritinophagy in paclitaxel-resistant breast cancer stem cells

**DOI:** 10.3389/fphar.2023.1201319

**Published:** 2023-09-28

**Authors:** Prasanthi Chittineedi, Santhi Latha Pandrangi, Juan Alejandro Neira Mosquera, Sungey Naynee Sánchez Llaguno, Gooty Jaffer Mohiddin

**Affiliations:** ^1^ Onco-Stem Cell Research Laboratory, Department of Biochemistry and Bioinformatics, School of Science, GITAM (Deemed to be) University, Visakhapatnam, India; ^2^ Department of Life Sciences and Agriculture, Armed Forces University-ESPE, Santo Domingo, Ecuador, South America

**Keywords:** cancer stem cells, ferritinophagy, mTOR, insulin, drug resistance

## Abstract

**Aim:** *Nyctanthes arbortristis Linn* is a potential anti-diabetic drug that reduces glucose levels by delaying carbohydrate digestion. The tumor microenvironment is characterized by elevated glucose levels that activate various genes, such as mTOR. mTOR plays a critical role in maintaining the hypoxic environment and inhibiting autophagy. Although natural compounds pose fewer side effects, their hydrophobic nature makes these compounds not suitable as therapeutics. Hence, we conjugated aqueous NAT into gold nanoparticles (AuNP) in the current study and evaluated the ability of the chosen drugs to induce cell death in breast cancer cells resistant to Paclitaxel.

**Materials and methods:** Particle size analyzer, UV-Vis spectrophotometer, FTIR, and XRD were used in the present study to characterize NAT and Doxorubicin encapsulated AuNPs. To check the cytotoxic effect of AuNP-NAT and AuNP-doxorubicin on Pac^R^/MCF-7 stem cells MTT assay was performed. RT-PCR was performed to check the altered expression of ferritinophagy-related genes. The proliferation and migration potential of the cells before and after treatment with the desired drug combinations was evaluated by clonogenic and scratch assays, respectively. Flow cytometry analysis was done to quantify apoptotic bodies and cell cycle arrest. Cellular ROS was determined using DCD-FA staining.

**Results and conclusion:** NAT and doxorubicin loaded into AuNP showed enhanced stability and induced ferritinophagy in Pac^R^/MCF-7 stem cells. The obtained results suggest that AuNP-NAT, combined with a low AuNP-Doxorubicin nanoconjugate dose, might be an effective anti-neoplastic drug targeting the necroptosis-autophagy axis, thereby reducing the adverse side-effects induced by the conventional chemotherapeutic drugs.

## Introduction

Cancer is the second leading cause for the rise in mortality rate all over the world contributing approximately 12% of the world’s mortality. Current treatment regimen such as surgical removal of tumor, chemotherapy, and radiation therapy could not show its impact on acquired resistance leading to tumor relapse/recurrence (Aldinucci et al., 2020). Although conventional therapies make patient’s cellular microenvironment tumor-free they pose major threat to the overall survival outcome of the patient due to their adverse side effects. Due to their minimal side-effects and better therapeutic potential plant derived drugs are being investigated.

*Nyctanthes arbortristis* (NAT) is considered a mythological plant possessing high medicinal values such as anti-helminthic and antipyretic, anti-cancer, anti-leishmania, anti-inflammatory, antiallergic, immunomodulatory, and antiviral (Latha Pandrangi et al., 2022). Although the anti-proliferative effects of this plant have been explored, to the best of our knowledge, there are no studies investigating the pathway to induce tumor cell death. Although very little is known about the mechanistic pathways in inducing tumor cell death, numerous studies showed NAT as a potent anti-diabetic drug. The active compound Arbortristoside-C extracted from NAT potentially inhibited mammalian α-glucosidase. Inhibition of *α*-glucosidase delays carbohydrate digestion and postprandial glucose absorption leading to insulin inhibition (Savic et al., 2016; Abdel-Wahab et al., 2019). It is evident that tumor cells show the Warburg effect, representing that these cells need a bulk amount of glucose, suggesting that NAT could induce tumor cell death by disrupting glucose homeostasis. Insulin resistance is one of the hallmarks of tumor progression. Hence targeting carbohydrate metabolism and/or insulin resistance might serve as a novel approach to cancer therapy.

Although phytochemicals are effective with minimal side effects as anticancer agents, they are not used clinically because of their hydrophobic nature and poor solubility (Pandrangi et al., 2022a). Although there are numerous ways to deliver the desired drugs to the targeted site to overcome the challenge, nanoparticle-based delivery of desired drugs serves to be more effective in delivering aquaphobic drugs to the target site, protecting them from degradation in blood as well as reducing dosage leading to low side-effects when compared with the conventional chemotherapy (Dong et al., 2014). AuNPs have gained more consideration as drug-delivering agents due to their benefits concerning surface characterization, which facilitates facile functionalization with both synthetic and biologically active compounds escorted by low toxicity to normal cells. Targeted therapy facilitates the meticulous drug release in precise quantities to the targeted sites. This results in augmentation of the drug efficacy and diminishes the overall drug dosage, ultimately resulting fewer side effects triggered by conventional drugs (Du et al., 2018). Das et al. suggested that ingesting Swarna-bhasma containing AuNPs showed a good prognosis and better survival outcomes in patients diagnosed with rectal cancer (Das et al., 2012). Hence, in the present study, we encapsulated crude NAT extract and commercial drug Doxorubicin and treated Pac^R^/MCF7 stem cells with an IC_50_ of AuNP-NAT and IC25 AuNP-Doxorubicin to induce iron-dependent cell death without altering the cytotoxic effect of Doxorubicin even at lower concentrations.

## Materials and methods

### Preparation and extraction of aqueous NAT

The leaves of the NAT were gathered, dried, and turned into powder. Methanol was used to extract the NAT powder. In a nutshell, the residue was wrapped in filter paper, deposited in the Soxhlet apparatus with methanol, and run until the reaction mixture became colorless. A rotary evaporator was used to collect and dry the colorless solution. Finally, 1 mg/mL concentration of NAT was prepared by dissolving the crude extract in UltraPure distilled water (Gibco) and was filtered using a 0.2 µ syringe filter and stored at −20°C until further analysis.

To get 5 mg/mL, 5 mg of Doxorubicin (Sigma) was diluted in 1XPBS (Gibco) and filtered through a 0.2 µ syringe filter and stored at 4°C until further analysis.

### Conjugation of the desired drugs with AuNPs

8 μg/mL concentration of AuNPs was prepared from the commercially purchased AuNPs (MBNPG001 HiMedia). To conjugate, the desired drugs, 3 mL of 8 μg/mL AuNP were combined individually with 1 mL of 1 mg/mL crude methanolic NAT extract and 1.2 μg/mL doxorubicin (3:1 ratio) and were stirred at room temperature overnight on a magnetic stirrer. The samples were ultracentrifuged for 10 min at 13,000 rpm the next day and was dissolved in nuclease-free water.

### Ultraviolet-visible (UV-Vis) spectroscopy

A double-beam UV-Vis spectrophotometer (Shimadzu UV-1800) was used to analyze the maximum absorption of the synthesized AuNPs and AuNPs encapsulated with NAT and doxorubicin nanocomposites. In brief, 3 mL of AuNP-drugs was transferred to a 1 cm UV-quartz cuvette, and absorption spectra was recorded.

### Fourier transform InfraRed spectroscopy analysis

To assess the functional groups contained in the nanoconjugates, FTIR measurements were taken with a Brucker FT-IR spectrometer at 500–4,500 cm^1^. In brief, 10 µL of samples were put on the probe and analysed to record the produced peaks.

### Zeta potential and particle size analysis

Zeta potential (ZP) representing the surface charge of the nanoparticles and particle size are the critical factors influencing the stability and capability of the conjugated nanoparticles to penetrate the nuclei, respectively. Hence, the ZP and size of both AuNP-NAT and AuNP-Doxorubicin were investigated using the Horiba SZ-100 and the photon correlation spectroscopy technique. The mean ZP was quantified with the help of a 60-s analysis time.

### Powder X-ray diffractometer (XRD)

The crystalline nature of the AuNPs loaded with NAT and Doxorubicin was evaluated utilizing a Brucker D8 advance X-ray Diffractometer with a copper cell. To detect the crystalline condition of the nanocomposites, the liquid samples were cast onto a microscopic slide that was cut into 1.5*1.5 cm dimensions, dried, and 2θ values were recorded in an XRD fitted with a Cu anode filter, and a 40kV/30mA D8 diffractometer.

### Determination of IC50 of paclitaxel, NAT, AuNP-NAT, AuNP-Dox, and dox

MCF-7 cells were plated in a 96-well plate and treated with paclitaxel, aq. NAT extract, AuNP-NAT, AuNP-Dox, and Dox at escalating concentrations for 48 h. Following incubation, cells were subjected to PBS wash and fed with 200 µL of drug-free media and 50 µL of MTT (Sigma-Aldrich). The cells were incubated for 3 h before being dissolved in 200 µL of DMSO. The optical density (OD) was recorded immediately at 570 nm using a ELISA plate reader (BIO-RAD). The OD obtained for the control cells was assumed to be viable, and the OD of the treated cells was computed accordingly. The IC_50_ value obtained from paclitaxel was utilized to develop paclitaxel resistance in MCF-7 clones, while the IC50 values of the remaining drugs were used further analysis.

### Cell line maintenance and development of Pac^R^/MCF-7

The MCF-7 cell line was obtained from the National Centre for Cellular Science in Pune. The cells were supplemented with 10%DMEM (Invitrogen), comprising Fetal Bovine Serum (FBS) (Gibco), 1% Glutamax (Invitrogen), and 1% Penstrep (Invitrogen). The cells were incubated in a 5% CO_2_ incubator until the flask was 90% confluent, then trypsinized and utilized to produce Pac^R^/MCF-7 stem cells, as previously reported (Chittineedi and Latha Pandrangi, 2022). In brief, 80% of confluent MCF-7 cells were incubated for 48 h with 30 nM paclitaxel. The next day, floating cells were removed, adherent cells were subjected to PBS wash to remove the excess drug, and allowed to grow until they were 80% confluent. Confluent cells were trypsinized again and subjected to a two-fold high dosage of paclitaxel drugs. This process was repeated at least ten times, and the MTT test was done on the drug-resistant clones that had emerged. MCF-7 cells without drug treatment were considered control cells.

### Analysis of stem cell markers in PacR/MCF-7 cells

To analyze the expression of various genes corresponding to stemness, FACS was performed. Briefly, the PacR/MCF-7 that were 80% confluent, were washed with ice-cold PBS twice and incubated with primary antibodies (OCT-4 and NANOG) for 30 min at 4°C. After incubation, the cells were washed and suspended in FITC-conjugated secondary antibodies. The cells were washed with ice-cold PBS and expression was analyzed using flow cytometry.

### Scratch assay

A wound healing test assessed the cellular migratory capability of Pac^R^/MCF-7 stem cells. Pac^R^/MCF-7 stem cells were plated in 35 mm Petri dishes and cultured at 37°C in a 5% CO2 incubator until 90% confluency. After a scratch across the center of the plate using 10 µL microtip, floating cells were removed and the cells were treated for 24 h with the IC_50_ NAT (50 μg/mL), IC_50_ AuNP-NAT (20 μg/mL) along IC_25_ AuNP-doxorubicin (0.9 μg/mL), and IC_50_ doxorubicin (3.7 μg/mL). As control cells without any drug treatment were chosen. The wound healing ability was checked for every 12 h, and photos were taken at a magnification of 4x.

### Clonogenic assay

A clonogenic experiment was performed to assess the capability of the effect of chosen drugs to inhibit colony formation. Briefly, Pac^R^/MCF-7 stem cells were trypsinized and plated on individual 35 mm Petri plates at a density of 1 × 10^3^ cells. The cells were treated with the desired concentrations (IC_50_ NAT (50 μg/mL), IC_50_ AuNP-NAT (20 μg/mL) along IC_25_ AuNP-doxorubicin (0.9 μg/mL), and IC_50_ doxorubicin (3.7 μg/mL)) and incubated for 24 h in a 5% CO2 incubator. As a control, drug-free Pac^R^/MCF-7 stem cells were used. After incubation, the plate was cleaned and supplied with drug-free 10%DMEM, and further incubated till the control plate formed colonies. After incubation, the cells were fixed with 70% ethanol and stained with 0.5% crystal violet and the colonies were captured using an inverted microscope at ×4 magnification (cilika). The number of colonies formed was further quantified using ImageJ software.

### Expression analysis using RT-PCR

To investigate the influence of NAT entrapped into AuNP on altering certain ferritinophagy markers’ gene expression, RT-PCR was performed. Pac^R^/MCF-7 cells were treated for 24 h in a 5% CO2 incubator with IC_50_ of NAT (50 μg/mL), IC_50_ of Doxorubicin (3.7 μg/mL), a combination of IC_25_ of AuNP-Doxorubicin (0.9 μg/mL), and IC_50_ of AuNP-NAT (20 μg/mL). Untreated cells served as the control. TriZol was used to extract RNA from both the treated and untreated groups, which was then converted to cDNA according to the manufacturer’s instructions (Abcam). Using primers and a fluorescent probe, obtained cDNA was utilized to examine the expression of several ferritinophagy makers (LC-IIIB, NCOA4, Ftn) (SYBR green) and was placed in a thermal cycler (Applied Biosystems). The obtained data was analyzed using LivakMode. (Table 1) represents the sequence of various primers used in the present study.

**TABLE 1:** Primers and their sequences used in the current study to analyze the expression of various genes.

Gene	Forward primer	Reverse primer
Ferritin	GCT​CTA​CGC​CTC​CTA​CGT​TT	GTG​GCC​AGT​TTG​TGC​AGT​TC
LC-3B	CAG​CGT​CTC​CAC​ACC​AAT​CT	GCG​GGT​TTT​GTG​AAC​CTG​AA
NCOA4 mRNA	GGG​CAA​CCT​CAG​CCA​GTT​AT	GGG​ATC​TGA​AAA​TTC​CCA​ACG​G
mTOR	CGCGAACCTCAGGGCAA	CTG​GTT​TCC​TCA​TTC​CGG​CT
GAPDH	ACA​GTC​AGC​CGC​ATC​TTC​TT	GGC​AAC​AAT​ATC​CAC​TTT​ACC

### Immunofluorescence

Immunofluorescence was performed to check the protein level of LC-3B in both treated and untreated groups of PacR/MCF-7 cells. Briefly, the cells were grown on coverslips and treated for 24 h with the respective drug concentrations (50 μg/mL for NAT, 20 μg/mL for AuNP-NAT, 0.9 μg/mL for AuNP-doxorubicin, and 3.7 μg/mL for doxorubicin). After incubation, the cells were fixed with 4% paraformaldehyde for 20 min, permeabilized with 02% triton-x 100 for 10 min, and blocked with 10% BSA for 30 min. Later the cells were incubated with the primary antibody (GTX127375) for 1 h at 37°C followed by secondary antibody incubation for further 1 h at 37°C. The cells were counter stained with 1 μg/mL DAPI, and the fluorescent images were captured in EVOS FLc fluorescent inverted microscope and quantified using ImageJ software. The cells were washed twice with 1X PBS after each step.

### Cell cycle analysis

Cell cycle arrest was assessed through flow cytometer. Briefly, Pac^R^/MCF-7 stem cells were treated for 24 h with chosen drug combinations (50 μg/mL for NAT, 20 μg/mL for AuNP-NAT, 0.9 μg/mL for AuNP-doxorubicin, and 3.7 μg/mL for doxorubicin) to study cell cycle arrest. To generate single-cell suspensions, both untreated and drug-treated cells were trypsinized with 1x-trypsin-EDTA (Gibco). The single-cell suspension was pelleted down and was washed twice in 1xPBS before being suspended in 50 µL of 100 μg/mL RNase and 200 µL of 50 μg/mL Propidium Iodide (PI). The mixture was incubated for 5 min before being examined with a flow cytometer (BD Acquri).

### Apoptosis assay

To analyze the percentage of cells that underwent cell death after drug treatment, cell apoptosis analysis using a flow cytometer was performed using an Annexin-V FITC Staining/Detecting kit (Abcam). Briefly, 80% confluent Pac^R^/MCF-7 stem cells were exposed to 50 μg/mL NAT, 20 μg/mL AuNP-NAT, 0.9 μg/mL AuNP-Doxorubicin, and 3.7 μg/mL Doxorubicin for 24 h, followed by trypsinization with 1X trypsin-EDTA (Gibco) to obtain single cell suspension. The single suspension cells were centrifuged, and the pellet was resuspended in 500 µL 1X binding buffer, 5 µL Annexin V-FITC, and 1 µL SYTOX green dye. The tubes were then incubated for 10 min at room temperature in a dark atmosphere. After incubation, the cells were analyzed.

### Cellular ROS assay

A high level of cellular ROS is a defining trait of a dying cell. As a result, a cellular ROS test was performed to confirm cell death. Pac^R^/MCF-7 stem cells were treated to the chosen medication concentration (50 μg/mL for NAT, 20 μg/mL for AuNP-NAT, 1.9 μg/mL for AuNP-doxorubicin, and 3.7 μg/mL for doxorubicin) and incubated for 24 h. Following that, drug-treated and control cells were stained for 30 min at dark in a humidified environment with 20 M DCF-DA (Sigma-Aldrich). Excess dye was removed by washing the stained cells with 1XPBS, and the cells were counterstained with nuclear stain (Hoechst 33342) for 5 min at dark to reveal cellular nuclei, followed by PBS washing to eliminate background noise. Microscopic pictures of the stained cells were captured under 4 × magnification using both DAPI and GFP filters in an inverted fluorescent microscope (EVOS FLc, Invitrogen). Captured pictures were quantified using ImageJ software.

### Statistical analysis

All tests were performed in triplicates, and the results are reported in terms of the mean. A Student’s t-test was executed to identify the differences. To determine the significance, Pearson coefficient was calculated. A *p*-value less than 0.05 was considered statistically significant.

## Results

### UV-vis spectrophotometer

**FIGURE 1:**
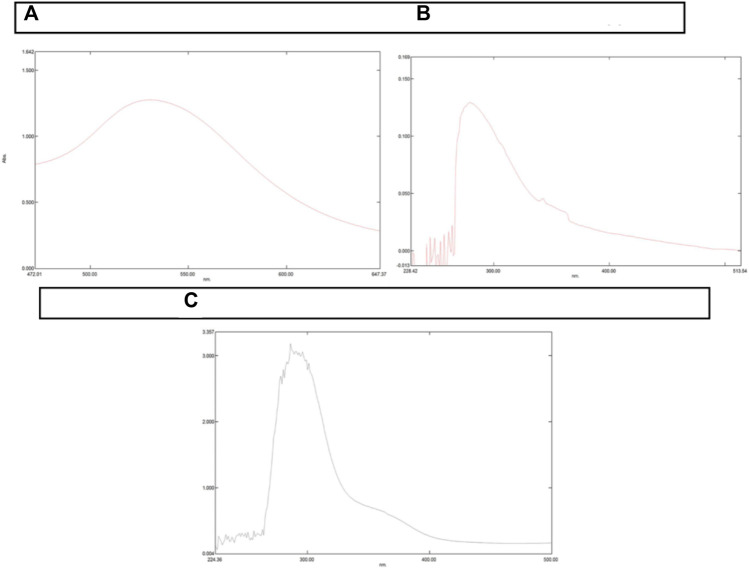
Graphical depiction of change in peaks resulted from UV-Vis Spectrophotometer. A UV-Vis Spectrophotometer is used to determine the maximum absorption of a certain substance and so forecast SPR. The peak shift indicates that a certain substance’s chemical makeup has changed. **(A)** illustrates *λ*_max_ of AuNP at 530 nm. **(B)** illustrates *λ*_max_ of AuNP-NAT with a shift in peak from 530 to 280 nm, while **(C)** represents *λ*_max_ of AuNP-Doxorubicin with a shift in peak from 530 to 280 nm. Because AuNPs were acquired commercially with *λ*_max_ at 280 nm, no AuNP analysis was performed.

UV-visible spectroscopy confirmed the loading of NAT and Doxorubicin to AuNPs. The present study employed AuNPs with maximum absorption of λmax ∼530 nm. As shown in Figure 1, *λ*_max_ of UV-Vis light of AuNP-NAT and AuNP-Doxorubicin were noticeably shifted from ∼530 to ∼280 nm and ∼525 nm, respectively, confirming that both drugs were possibly conjugated onto the AuNP surface, indicating that their photo-physical characteristics were altered. The width and site of the absorption peak would be contingent on various factors, for instance, their particle agglomeration, dielectric environment, particle morphology, and occasionally due to the 2° metabolites which are accountable for nanoparticle preparation regarded as Surface Plasmon Resonance (SPR).

### FTIR

**FIGURE 2:**
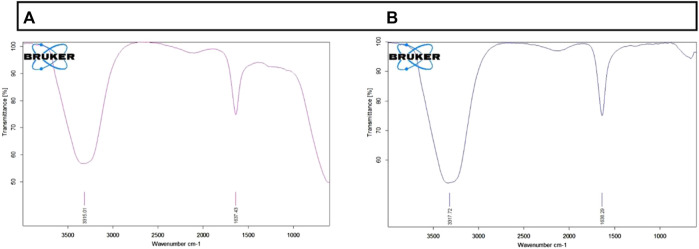
Graphical depiction of functional groups present in AuNP-NAT and AuNP-Doxorubicin. To identify the presence of functional groups after drug loading, FTIR analysis was performed. The above figure indicates that the chosen drug combinations were efficiently conjugated into the nanoparticles. **(A)** depicts functional groups present in AuNP-NAT; **(B)** depicts functional groups present in AuNP-Doxorubicin.

FTIR analysis plays a critical role in identifying the presence of functional groups in drug-conjugated nanoparticles. The spectrum of AuNP-Doxorubicin and AuNP-NAT that has been recorded between 3,000 and 300 cm^−1^ spectral region is shown in Figure 2. The FTIR spectrum of AuNP-NAT and AuNP-Doxorubicin displayed a symmetric stretching vibration frequency to the O-H phenol group at 3,317.72/cm for AuNP-doxorubicin and 3,315.01/cm for AuNP-NAT, respectively, suggesting alcoholic group. Additionally, the incidence of the N-H bond of 1° amines was logged at 1,638.29/cm for AuNP-doxorubicin and 1,637.43/cm for AuNP-NAT, respectively, representing the amide group. According to the results, both amide and phenol functional groups of the desired drug combinations shield AuNP from agglomerating, thereby enhancing AuNP stability.

### Zeta potential and particle size analysis

**FIGURE 3:**
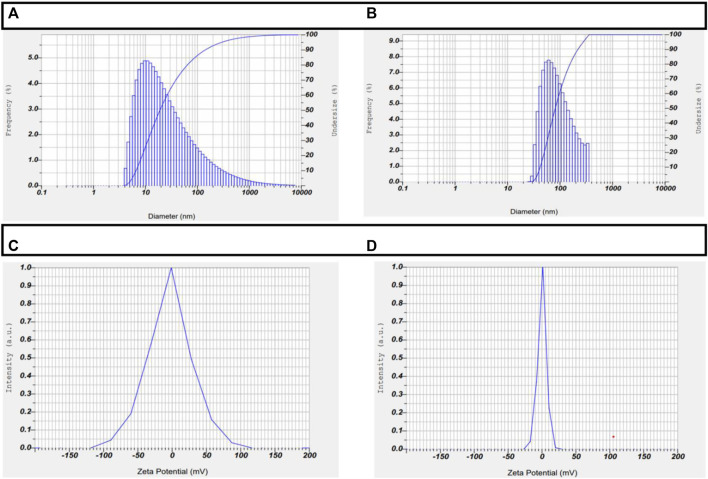
Depiction of ZP and particle size of AuNP-NAT and AuNP-Doxorubicin. DLS was used to perform ZP and particle size analyses on the drug-loaded nanoparticles to ensure their stability and particle size. The obtained results demonstrate that AuNP-NAT and AuNP-Doxorubicin have 2.7 nm and −3.7 mV, 1.9 nm, and +0.2 mV 2.7 nm particle size and particle size and 0.2 mV particle size and zeta potential, respectively, indicating that the drug-loaded AuNPs are very stable and proficiently enters the nuclei. **(A)** depicts AuNP-NAT particle size; **(B)** depicts AuNP-Doxorubicin particle size; **(C)** depicts AuNP-NAT Zeta potential; and **(D)** depicts AuNP-doxorubicin Zeta potential.

ZP and Particle size are 2 crucial properties of any nanoparticles that govern their ability to enter the nucleus and their subsequent stability within the cell. A zeta sizer was used to assess the size distribution and surface charge of the drug-loaded AuNPs. AuNP-NAT and AuNP-Doxorubicin had average particle sizes and zeta potentials of 2.7 nm and −3.7 mV, 1.9 nm, and +0.2 mV, respectively. The surface stability of the encapsulated NAT and doxorubicin has been demonstrated (Figure 3).

### Powder-X ray diffractometer (XRD)

**FIGURE 4:**
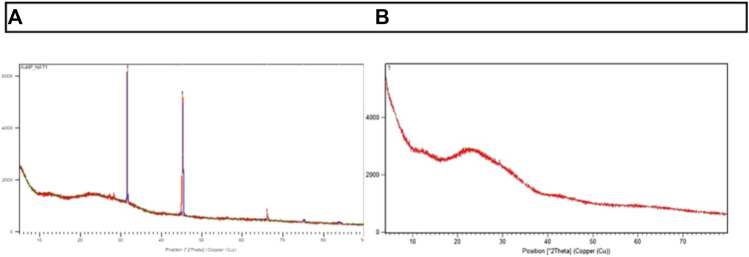
Representation of Powder XRD peaks of AuNP-NAT and AuNP-Doxorubicin. The crystalline nature of the nanoconjugates was determined using powder-XRD, and 2θ values were attained. **(A)** displays the peaks of x-ray diffraction in AuNP-NAT, whereas **(B)** displays the peaks of x-ray diffraction in AuNP-Doxorubicin.

Powder XRD was performed to identify the crystalline nature of the entrapped AuNPs (AuNP-NAT and AuNP-Doxorubicin) As shown in Figure 4, AuNP-NAT exhibited two intense peaks at 31.50 and 45.30, respectively, with minor peak at 66.090, 75.190, and 83.850. In contrast, AuNP-Doxorubicin exhibited one intense peak at 21.70, with numerous minor peaks, suggesting that the entrapped drugs possess a cubic structure with poly-crystalline faces.

### Development of paclitaxel resistance breast cancer cells

Breast cancer cells were treated with various concentrations of paclitaxel for around ten cycles to form Pac^R^/MCF-7 stem cells, and the cytotoxicity of paclitaxel was assessed using an MTT test after each cycle. Surprisingly, no further rise in the IC_50_ of breast cancer cell lines was observed after the sixth cycle, implying that the cells attained resistance towards paclitaxel at 90 nM concentration, which is three times higher than that of the IC_50_ of parenteral cell lines, implicating a three-fold increase in the IC50 of paclitaxel (Supplementary Data).

### Determination of IC50

To assess the IC50 in the cells, an MTT experiment was done on Pac^R^/MCF-7 stem cells using NAT, AuNP-NAT, AuNP-doxorubicin, and doxorubicin. The total numb. of viable cells fell steadily with a rise in the concentration of the desired drugs. A standard graph plotted with an MTT reagent concentration *versus* OD at 570 nm was used to estimate the IC_50_ value. Obtained absorbance was used to compute the concentration of the chosen drugs. Pac^R^/MCF-7 stem cells showed an IC_50_ of 50 μg/mL for NAT, 20 μg/mL for AuNP-NAT, 1.9 μg/mL for AuNP-doxorubicin, and 3.7 μg/mL for doxorubicin, indicating that loading of NAT and Doxorubicin into AuNPs lowered the IC_50_ values of the respective drugs.

### Stem cell expression analysis

**FIGURE 5:**
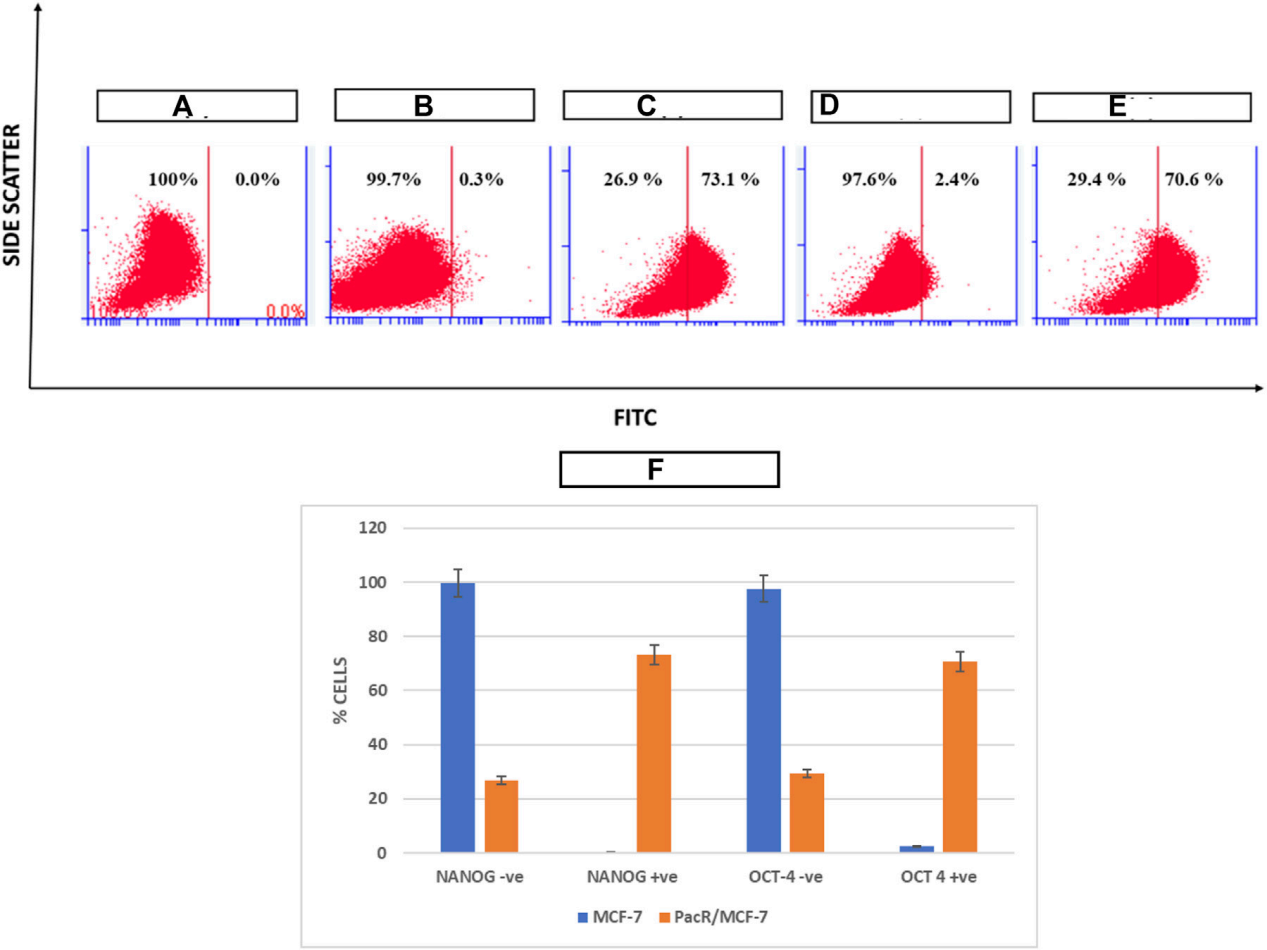
Pictorial representation of expression of NANOG and OCT4 in PacR BCSCs. NANOG and OCT4 are among the stem cell markers that determine the self-renewal ability and proliferation of CSCs. **(A)** represents unstained MCF-7 cells; **(B)** represents expression of NANOG in MCF-7 cell lines stained with FITC; **(C)** represents expression of NANOG in PacR/MCF-7 cells. **(D)** represents expression of OCT-4 in MCF-7 cell lines stained with FITC; **(E)** represents expression of OCT-4 in PacR/MCF-7 cells. **(F)** represents the bar graph of % cell variation in stem cell markers in both MCF-7 and PacR/MCF-7 cells. MCF-7 cells exposed to increased concentration of paclitaxel showed a drastic increase in the expression of both the markers from 0.3% to 73.1% for NANOG and from 2.4% to 70.6% for OCT-4, respectively.

OCT-4 and NANOG are the major drivers of self-renewal ability in cancer stem cells. Hence expression of these markers was analysed using Flow Jo software in drug resistant clones in order to evaluate the stemness potential. As shown in Figure 5, MCF-7 cells exposed to increased concentration of paclitaxel showed a drastic increase in the expression of both the markers from 0.3% to 73.1% for NANOG and from 2.4% to 70.6% for OCT-4 respectively suggesting that the acquired drug resistance lead to the increase in stemness of MCF-7 cell lines.

### Wound healing assay

**FIGURE 6:**
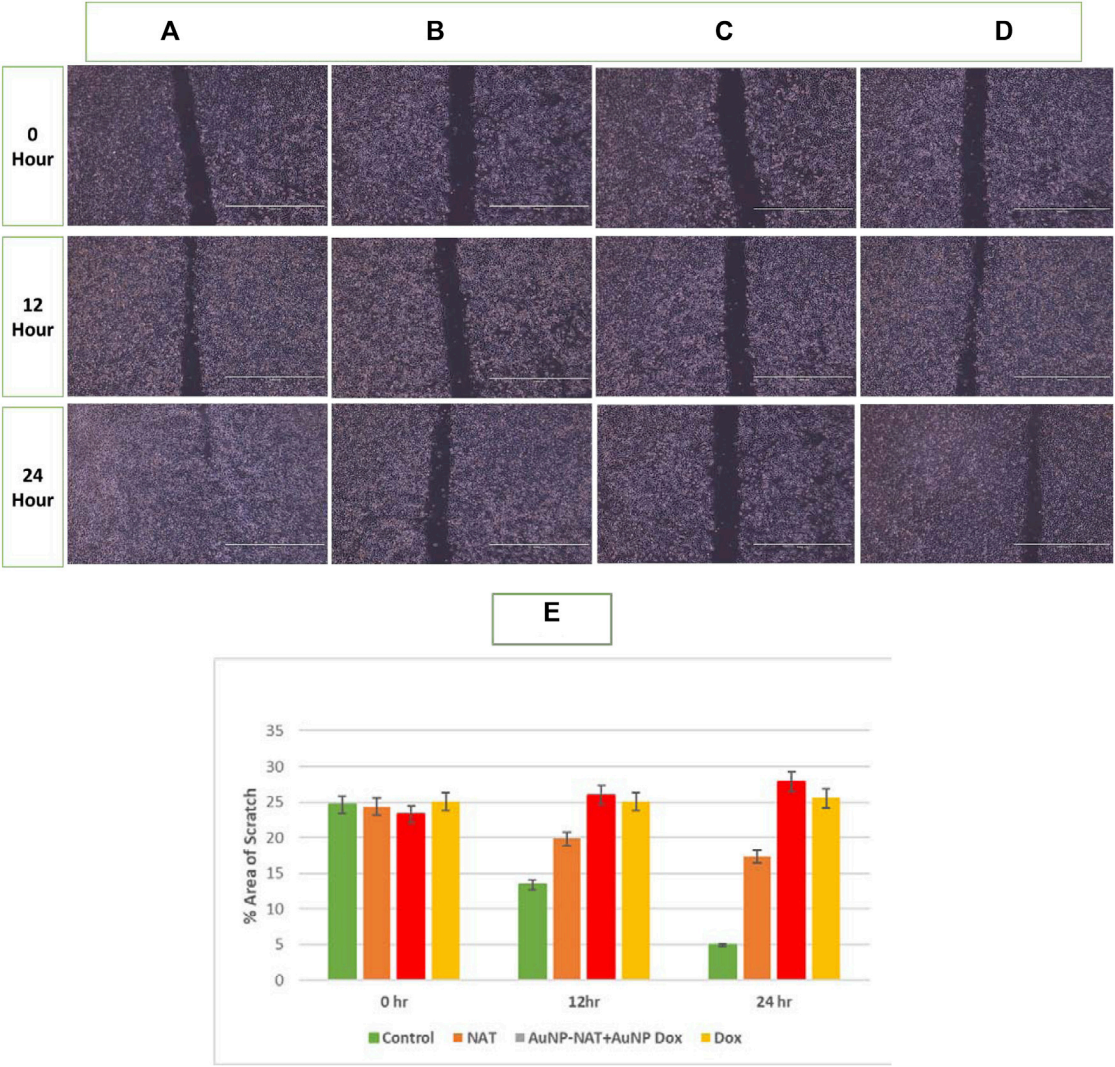
Pictorial representation of migration potential of Pac^R^/MCF-7 cell lines. A scratch was made using a scrapper in 35 mm plated with 90%–100% confluent cells. The cells were subsequently treated for 24 h with NAT **(B)**, AuNP-NAT in conjunction with AuNP-Doxorubicin **(C)**, and Doxorubicin **(D)**. As a control, untreated cells **(A)** were used. The wound was practically closed in control cells. Still, a constant breadth of the wound was detected in cells treated with chosen drugs, indicating that the indicated drugs likely reduced PacR/MCF-7 stem cell metastasis. **(E)** represents the % area that has been healed after treatment which is quantified by ImageJ software. As shown in the graph, combination of AuNP-NAT and AuNP-Dox effectively inhibited the wound closure (% area is relatively unchanged) compared with NAT and Dox alone suggesting that AuNP-NAT in combination with low concentrations of AuNP-Dox could potently inhibit cell migration.

Malignant cells are capable of moving and infiltrating different tissues, which indicates its hostile nature and migratory capability. A powerful anti-tumor medicine is believed to limit tumor migration, resulting in less tumor spread. A scratch was produced with a scraper to evaluate the ability to invade, and these cells were subjected to the chosen drug concentrations. As a negative control, untreated cells were used. The width of the scratch was evaluated under a microscope after exposure to the desired drugs. As depicted in the Figure 6, wound closure is not observed even after 24 h after drug treatment with desired concentrations when compared with the control, which was almost restored within 24 h, indicating that the desired drug possibly halted Pac^R^/MCF-7 stem cells metastatic ability.

### Clonogenic assay

**FIGURE 7:**
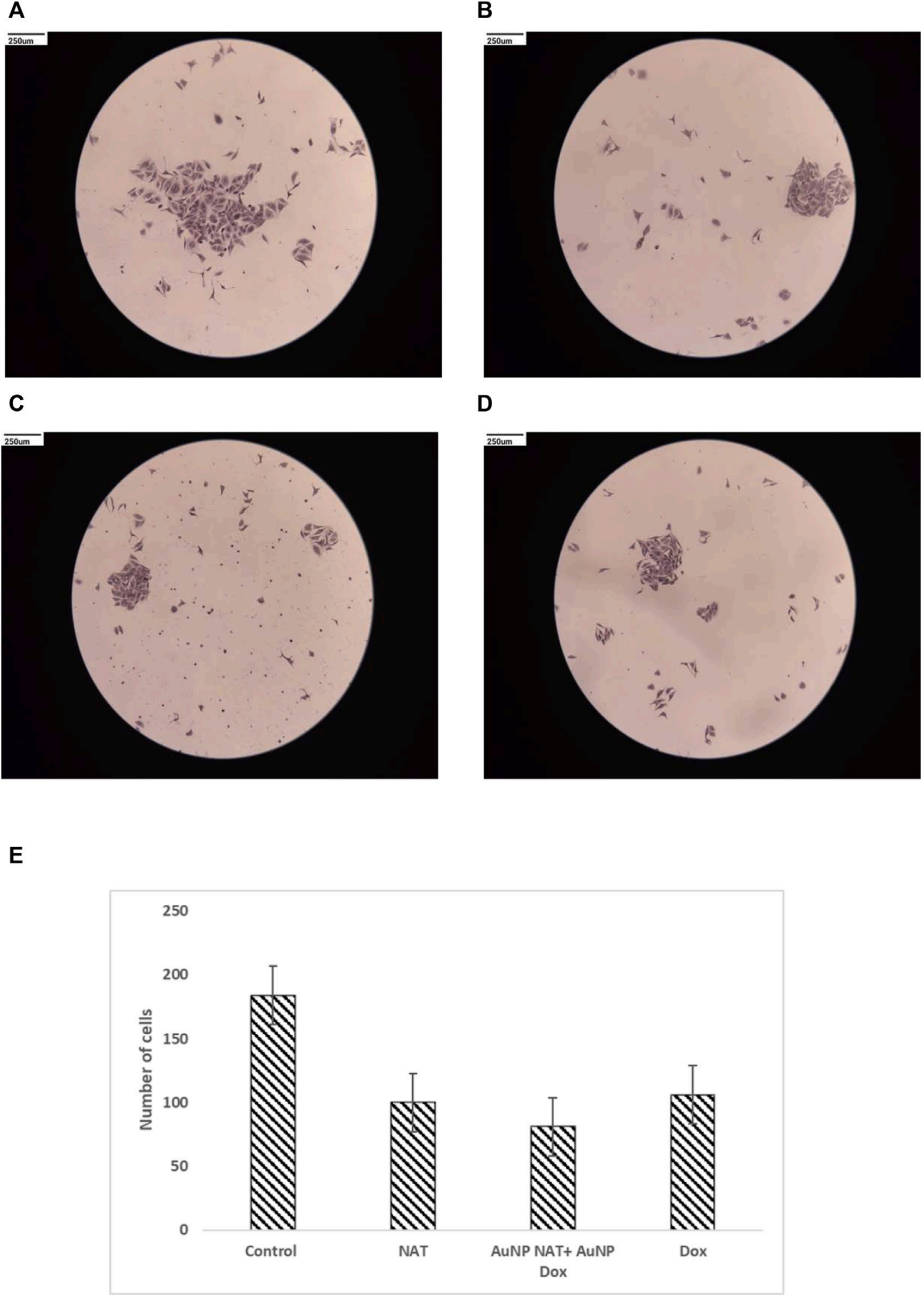
Pictorial representation of clonogenic ability of PacR/MCF-7 cell lines. Individual cells were seeded and incubated for 24 h in 35 mm Petri dishes. The cells were exposed for about 24 h to NAT **(B)**, AuNP-NAT in conjunction with AuNP-Doxorubicin **(C)**, and Doxorubicin **(D)**. As a control, untreated cells **(A)** were used. Finally, the cells were observed and the number of colonies produced was reported. The picture shows that all combinations might inhibit colony development, reducing tumor-initiating capabilities. **(E)** is the graphical representation of number of cells that was quantified using ImageJ software.

To evaluate the impact of the chosen drug concentration in inhibiting the formation of colonies, single cells were plated and exposed to the chosen drugs for 24 h in 35 mm Petri plates. Untreated cells were the negative control, whereas Doxorubicin was the positive control. The cells’ capacity to form colonies was tested after medication treatment. As predicted, NAT, Doxorubicin alone, and AuNP-NAT in conjunction with AuNP-Doxorubicin all could reduce colony formation (Figure 7).

### Analysis of ferritinophagic gene markers using RT-PCR

**FIGURE 8:**
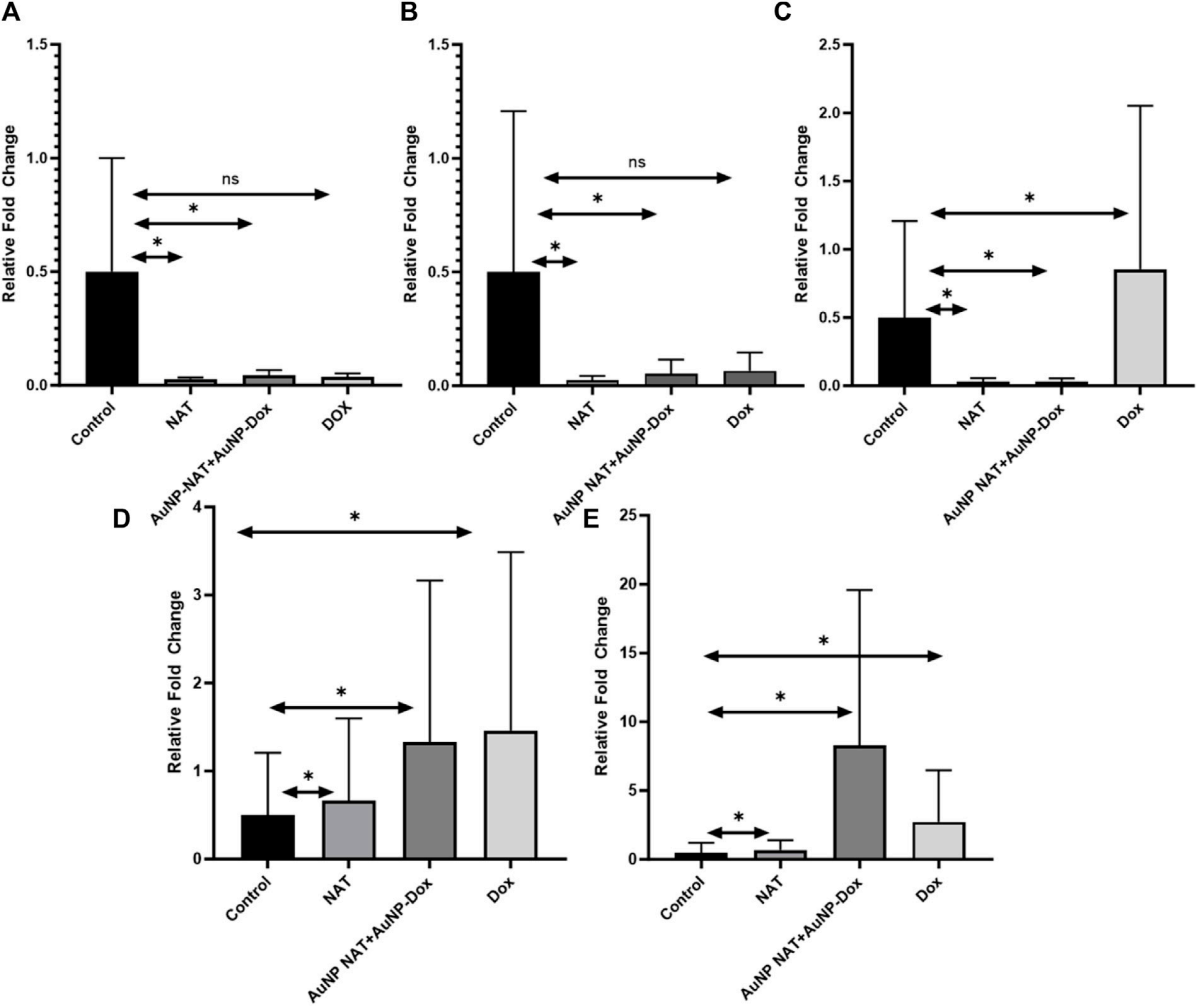
RT-PCR analysis of ferritinophagic specific markers. RT-PCR amplifies the gene of interest using specific primers. In **(A)**, the graph illustrates the relative gene expression of mTOR. **(B)** showcases the relative gene expression of Insulin. In **(C)**, the graph demonstrates the relative gene expression of Ferritin. **(D)** represents the relative gene expression of NCOA4. Lastly, **(E)** exhibits the relative gene expression of LC-3B. The obtained results reveal a notable suppression of carcinogenic ferritinophagy markers. Conversely, there is a significant elevation in the levels of ferritinophagy tumor suppressor markers. (Data represents ±SEM, by one-way ANOVA, *p < 0.05, n. s = not significant).

To examine the regulation of various ferritinophagy related markers after treating Pac^R^/MCF-7 stem cells with desired drug concentrations (IC_50_ AuNP-NAT, IC_25_ AuNP-Doxorubicin, IC_50_ NAT, and IC_25_ Doxorubicin, IC_50_ Doxorubicin), RT-PCR was performed. RT-PCR analysis revealed that the negative modulators of autophagy and ferroptosis, i.e., mTOR and ferritin, were suppressed, with associated upregulation of LCIIIB, a positive modulator of ferritinophagy. As cited in the literature, NAT suppressed insulin gene expression as well suggesting the correlation between insulin gene expression and induction of ferritinophagy (Figure 8). However, statistical analysis suggests that relative fold change of mTOR and Insulin treated with Dox showed non-significant correlation with *p*-value greater than 0.05 when compared to the untreated group.

### Immunofluorescence

**FIGURE 9:**
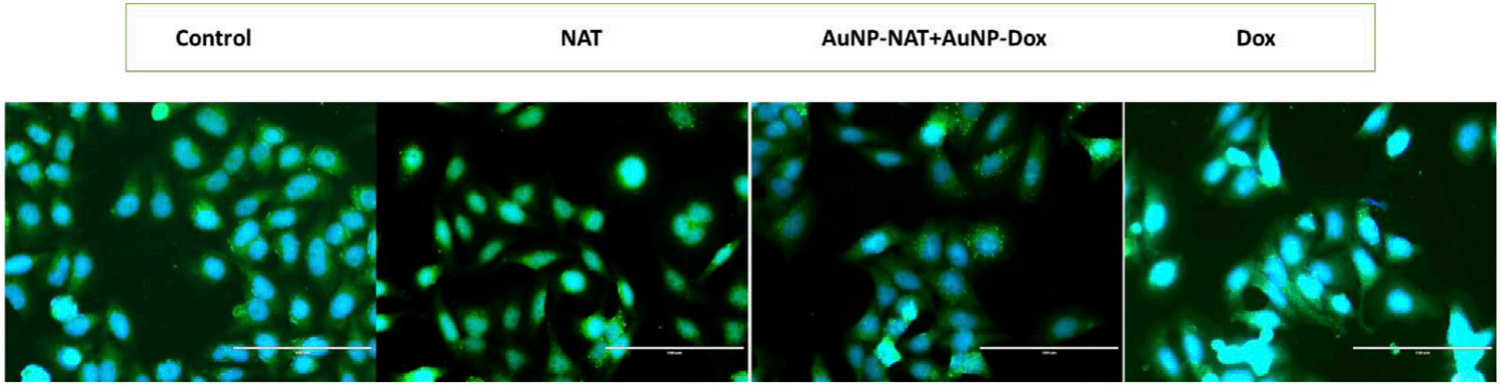
Fluorescent images representing LC-3B puncta formation in PacR/MCF-7 cells treated with the chosen drugs. LC-3B, an important marker that plays a crucial role in autophagy, is analysed by immunofluorescence. As shown in the figure the treatment groups induced puncta formation representing the activation of autophagic flux. The green fluorescence represents LC-3B, while the blue fluorescence represents the nuclei.

LC-3B can degrade many proteins, including ferritin, and is one of the reliable markers for autophagy. Gene expression analysis suggests that LC-3B and NCOA4, crucial to ferritinophagy induction, are markedly increased in the treated group compared to untreated control. To corroborate the gene expression results, the expression of LC-3B was checked by immunofluorescence in both treated and untreated groups. As shown in Figure 9, treated cells showed puncta formation with a high number of puncta in combined treatment of AuNP-NAT and AuNP-Dox compared to other treatments indicating that the combination of AuNP-NAT and AuNP-Dox drastically induced autophagic flux, degraded ferritin leading to the induction of ferritinophagy.

### Cell cycle analysis

**FIGURE 10:**
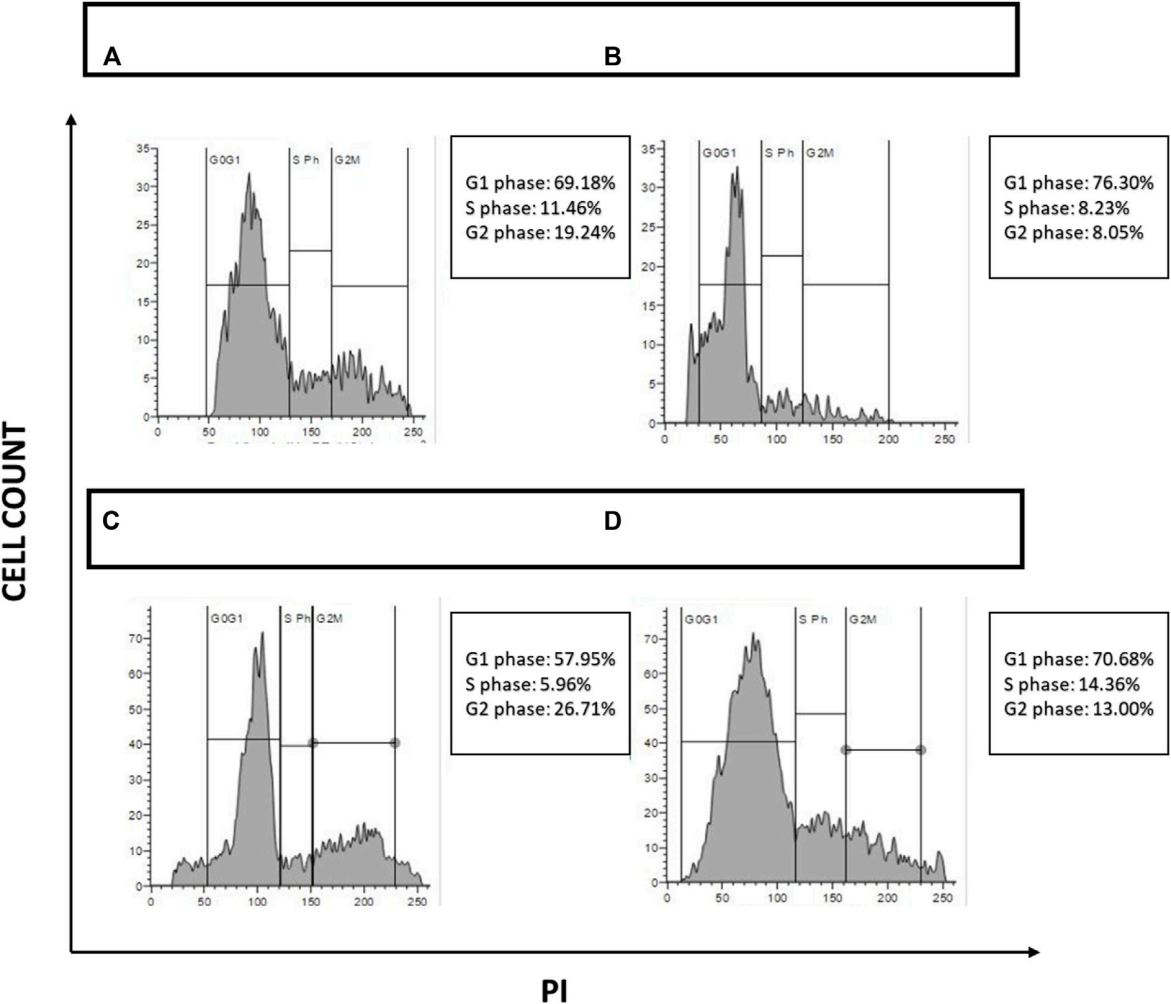
Depiction of cell cycle analysis acquired through FACS. Figure 10 depicts cell cycle analysis and their quantification. **(A)** Pac^R^/MCF-7CSCs under control; **(B)** Pac^R^/MCF-7 CSCs treated with NAT IC_50_ alone; **(C)** Pac^R^/MCF-7 CSCs treated with a combination of AuNP-NAT IC_50_ and AuNP-doxorubicin IC_25_; and **(D)** Pac^R^/MCF-7 CSCs treated with doxorubicin IC_50_ alone.

Tumor cells exhibit erratic cell proliferation due to different growth factors that are intrinsically mutated. Novel antineoplastic drugs are distinguished by their capacity to block cell cycle progression at checkpoints, therefore sensitizing malignant cells to cell death. The impact of the chosen drugs on cell progression has been assessed to investigate further the effects of NAT, AuNP-NAT in combination with AuNP-Doxorubicin, and Doxorubicin inducing ferritinophagy. Pac^R^/MCF-7 stem cells were treated with ribonuclease followed by PI staining, and the passage of cells through various cell cycle stages was observed and the obtained data was analysed using Flow Jo software. The results show that NAT alone induced cell cycle arrest in the G1 phase, whereas combination of AuNP-NAT and AuNP-Dox halted cellular proliferation by arresting the cells at G2/M phase. In contrast, Dox induced the cell cycle arrest at the S-phase, suggesting that a combination of AuNP-NAT and AuNP-NAT could potentially target the proliferation of PacR/MCF-7 clones (Figure 10).

### Cell apoptosis assay

**FIGURE 11:**
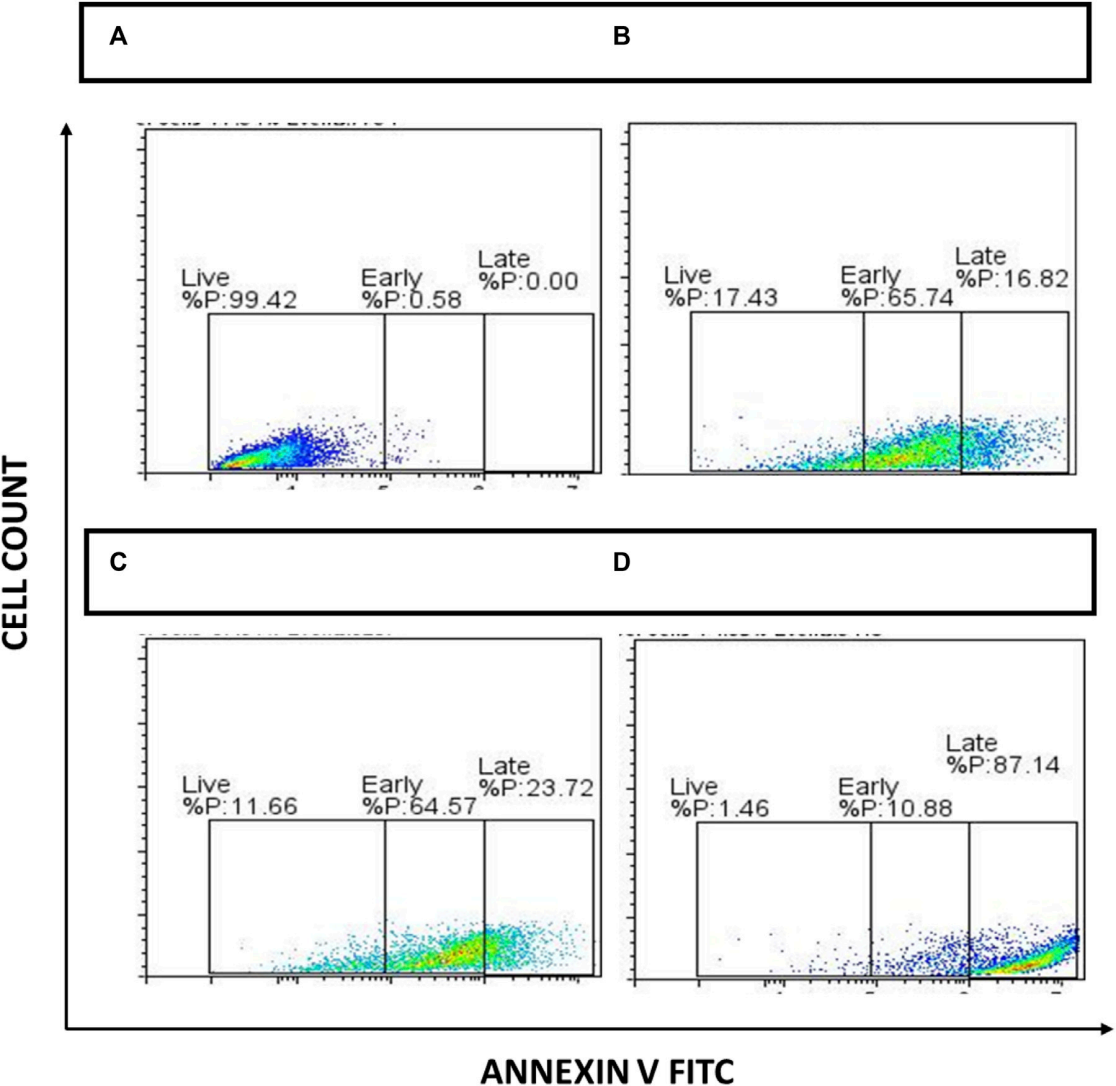
Depiction of apoptosis analysis acquired through FACS. During 24 h, the cells were treated to NAT **(B)**, AuNP-NAT in conjunction with AuNP-Doxorubicin **(C)**, and Doxorubicin **(D)**. Untreated cells were used as controls **(A)**, and drug-treated cells were exposed to Annexin V FITC apoptosis kit as directed by the manufacturer. Lastly, FACS Acquri was used to track cell death.

To further analyze the apoptotic cells generated, apoptosis analysis was performed using Annexin-V FITC staining/detection kit as per manufacturer’s instructions. After exposure of cells to 50 μg/mL of NAT, 20 μg/mL AuNP-NAT in combination with 1.9 μg/mL AuNP-Doxorubicin, and 3.7 μg/mL Doxorubicin, the percentage of apoptotic cells was analyzed using Flow Jo software. Obtained data suggests PacR/MCF7 stem cells treated with the chosen drugs significantly increased apoptotic cell populations. The viable PacR/MCF7 cells have been decreased from 99.43% to 17.43%, 11.66%. and 1.46% in cells treated with NAT, AuNP-NAT in combination with AuNP-Doxorubicin, and Doxorubicin respectively. However, a pronounced elevation from 0.58% to 65.74%, 64.57%, and 10.88% in the annexin-V+/SYTOX-quadrant representing early apoptosis for the cells treated with NAT, AuNP-NAT in combination with AuNP-Doxorubicin, and Doxorubicin respectively, while a gradual reduction from 0.00% to 16.82%, 23.72%, and 87.14% has been observed in the annexin-V+/SYTOX + quadrant representing late apoptosis for the cells treated with NAT, AuNP-NAT in combination with AuNP-Doxorubicin, and Doxorubicin respectively. The obtained results suggest that NAT and AuNP-NAT in combination with AuNP-Doxorubicin, induced early apoptosis, while Doxorubicin induced late apoptosis (Figure 11).

### Cellular ROS assay

**FIGURE 12:**
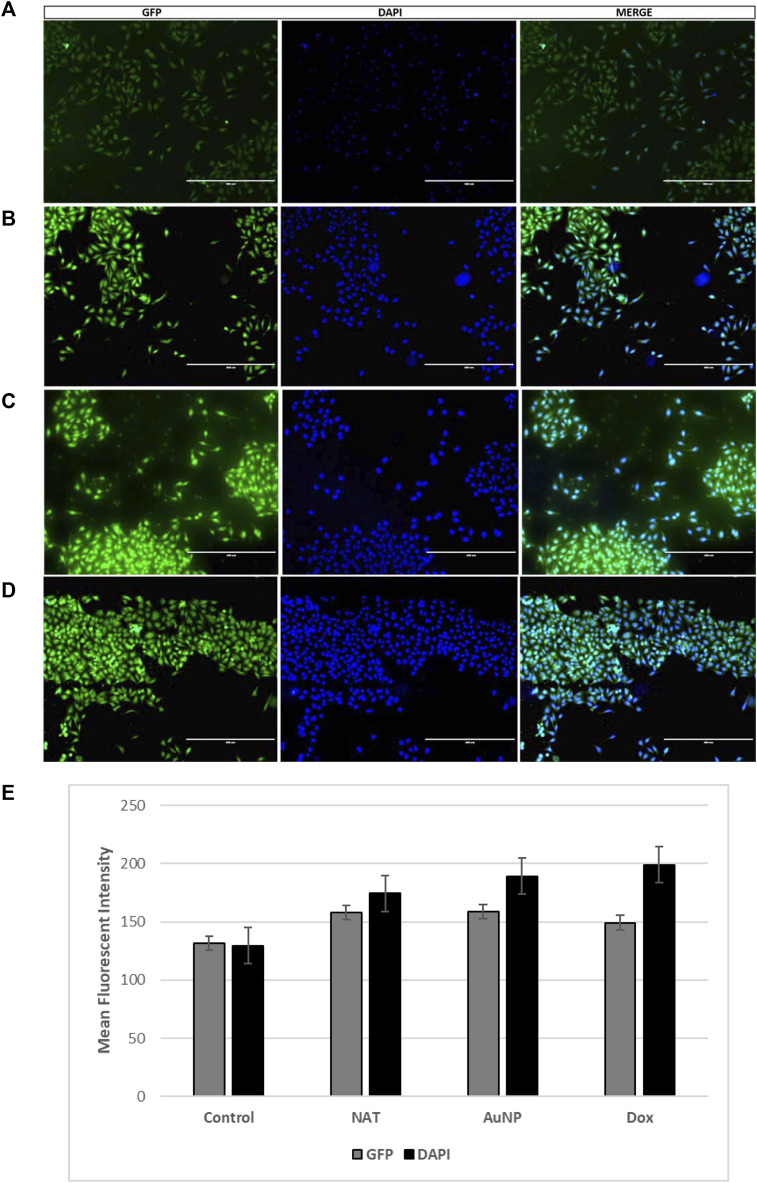
Fluorescent microscopic images of cellular ROS. Briefly, the cells were treated for 24 h with NAT **(B)**, AuNP-NAT in conjunction with AuNP-Doxorubicin **(C)**], and Doxorubicin **(D)**. Control cells **(A)** were chosen because they were untreated. The fluorescence intensity of the stains quantified using ImageJ software is shown graphically in **(E)**. (*p* < 0.05).

Mitochondrial disruption is a distinguishing feature of tumor cell sensitization to cell death mechanisms. To govern whether the chosen drugs resulted in the accumulation of cellular ROS, thereby leading to the antiproliferative effect of Pac^R^/MCF-7, ROS levels were measured using DCF-DA fluorescent staining. DCF-DA, which specifically stains and to visualize cellular ROS. DCF-DA detection of cellular ROS is dependent on diffusion. Once within the cell, DCF-DA is deacetylated to generate a non-fluorescent molecule governed by cellular esterase. When this chemical combines with ROS, it produces 2′,7′dichlorofluorescein (DCF) with high fluorescent intensity. Cellular ROS was quantified by the intensity of the fluorescence emission. The images of stained cells were captured at 10X magnification using DAPI and GFP filters in an inverted fluorescent microscope and the fluorescence intensity was quantified using ImageJ software. Figure 12 shows Pac^R^/MCF-7 stem cells treated with the chosen drug combinations exhibiting pyknotic nuclei compared to control cells, demonstrating that both the treatments alone and combined induced ROS formation.

## Discussion

Due to the adverse side effects, development of resistance to various therapies, and increase in the incidence of tumor relapse/recurrence, investigating in the therapeutic role of various compounds is necessary. Because of the inevitable nature of plant extract to heal various diseases, the isolation of active compounds from plants that are rich in anti-oxidants has gained more attention. Hence, exploring nature to find phytochemicals that show anti-cancer effects is important. *Nyctanthes arbortristis* is one among them, which has a wide range of applications in the medicinal field. Among various diseases, NAT extracts and their active compounds greatly impact regulating the blood glucose levels (Khanapur et al., 2014). Studies demonstrated that the purified compound of NAT serves as a mammalian α-glucosidase inhibitor which regulates endothelial cell glycosylation, angiogenesis, and tumor cell proliferation.

As an *α*-glucosidase inhibitor, NAT extracts could regulate glucose levels by delaying carbohydrate digestion. Interestingly, synthetic *α*-glucosidase inhibitors such as acarbose showed anti-cancer properties and abridged the incidence of colorectal cancer in a dose-dependent manner in diabetic patients (Dirir et al., 2022). However, the exact mechanism through which the *α*-glucosidase inhibitor has induced tumor cell death has not been demonstrated. To the best of our knowledge, this is the first study to investigate the underlying mechanism of natural *α*-glucosidase inhibitors in inducing tumor cell death. With the available literature, we hypothesized that since *α*-glucosidase inhibitors could inhibit carbohydrate digestion, thereby reducing glucose levels, tumor cells might undergo glucose starvation leading to death.

Mounting evidence suggests that glucose is one of the key regulators for activating the mTOR signaling pathway. mTOR monitors the growth and metabolism of eukaryotic cells by providing environmental inputs to deliver nutrients and growth factors (Morita et al., 2015; Saxton and Sabatini, 2017; Roy et al., 2022). mTOR regulates many elementary cellular processes, such as protein synthesis and autophagy. Additionally, mTOR induces HIF-1α expression in the tumor microenvironment, thereby maintaining a hypoxic environment (Morita et al., 2013; Pérez-Plasencia et al., 2019). Both HIF-1α activation and autophagy inhibition play a critical role in degrading ferritin (Choi et al., 2009; Huang and Zhou, 2020). This iron storage protein that is the key regulator in inducing ferritinophagy, a novel cell death pathway that is mediated through autophagic degradation of ferritin (Chittineedi et al., 2022).

NCOA4 is a cargo protein marker for ferritinophagy induction, associated with iron homeostasis regulation. This protein regulates intracellular iron levels by binding to ferritin and subjecting it to autophagic degradation under iron deprivation, which results in ferritinophagy (Alkhateeb and Connor, 2013; Ryu et al., 2018). While NCOA4 depletion inhibits ferritin delivery into the lysosome, which results in a significant decrease in bioavailable intracellular iron (Pandrangi et al., 2022b; Pandrangi et al., 2022c). This cargo protein is referred to as the autophagy receptor for ferritin by Dowdle et al. RT-PCR analysis revealed that upon treatment with NAT and Doxorubicin individually and in combination significantly reduced the expression of ferritin associated with significant upregulation of NCOA-4 and LC-IIIB gene, and immunofluorescence study revealed a significant increase in autophagic flux indicated by puncta formation suggesting that the desired drug combinations induced ferritinophagy through downregulating mTOR signaling cascade. Downregulation of mTOR leads to the inactivation of HIF-1α, thereby reducing the hypoxic conditions in the hostile microenvironment.

Despite these advantages, phytochemical-based anti-cancer treatment is rarely used in clinical trials due to its low solubility. To address this issue, many medication delivery methods have been developed (Pandrangi et al., 2022d). A nanoparticle-based drug delivery method, for example, is more competent in delivering aquaphobic drugs into cells, protecting them from degradation in bloodstream. AuNPs have gained popularity as drug delivery vehicles due to their advantages in surface characterization, which allows for facile functionalization with biological and synthetic chemicals while remaining low in toxicity (Rajesh, 2000; Rambatla et al., 2021). The meticulous release of the antineoplastic drugs in precise proportions at the targeted areas is made possible by targeted drug delivery. This strategy improves the drug’s efficacy and decreases the overall dose, resulting in unanticipated adverse effects induced by anti-cancer compounds.

This work aims to encapsulate the isolated NAT component into AuNPs and test its therapeutic effect when combined with modest concentrations of AuNPs loaded with doxorubicin. The commercial drugs Doxorubicin and NAT alone were used as positive controls to confirm the results. Characterization investigations demonstrate that NAT and doxorubicin loaded into AuNPs have superior stability, with ZPs of −3.7 and 0.2 mV, respectively, and particle sizes of 2.7 and 1.9 nm. According to the cited literature, AuNPs loaded with diverse drugs have high stability with a ZP ranging from +30 to −30 mV. Moreover, the research reveals that AuNPs with smaller particles than 50 nm can easily infiltrate the nuclei and exhibit cytotoxicity on DNA. Cell cycle studies revealed that AuNP-NAT in combination with AuNP-doxorubicin might induce G2/M-phase arrest, which indicates inhibition of cell proliferation. These findings indicate that the loaded medicines may have entered the nucleus and caused DNA damage. RT-PCR expression profiling was performed to analyze the expression of ferritinophagy genes and corroborated the results by performing immunofluorescence to check the puncta formation a characeteristic feature of cell during enhanced autophagic flux to better explain the mechanism by which the chosen compounds produced cell death.

## Data Availability

The raw data supporting the conclusion of this article will be made available by the authors, without undue reservation.
