# Supplementary material for: Aqueous Nyctanthes arbortristis and doxorubicin conjugated gold nanoparticles synergistically induced mTOR-dependent autophagy-mediated ferritinophagy in paclitaxel-resistant breast cancer stem cells
Source: Front Pharmacol. 2023 Sep 28;14:1201319. doi: 10.3389/fphar.2023.1201319 (PMC10568009; doi:10.3389/fphar.2023.1201319)
Supplement: Supplementary file 1 [file DataSheet1.DOCX]

**
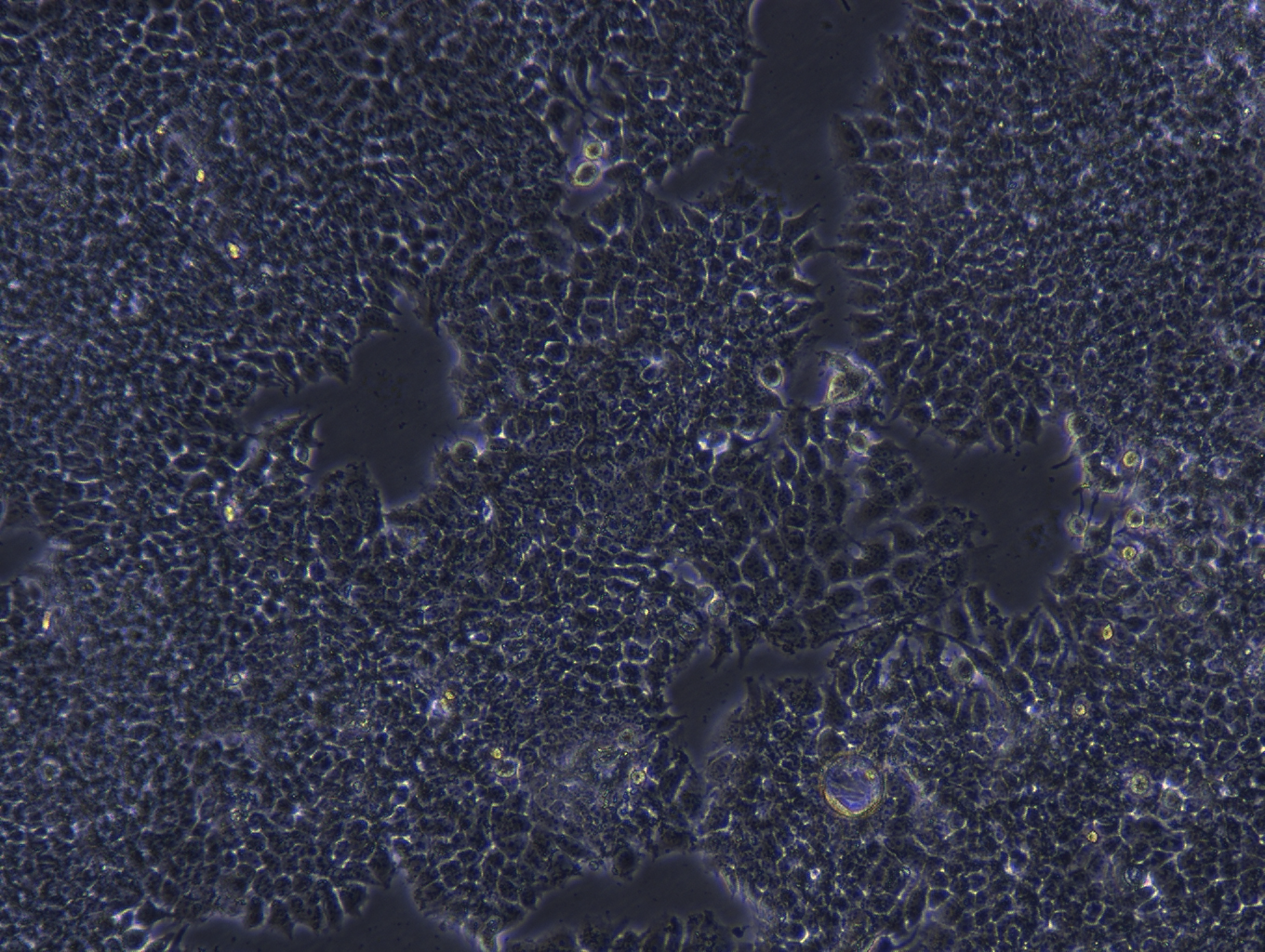

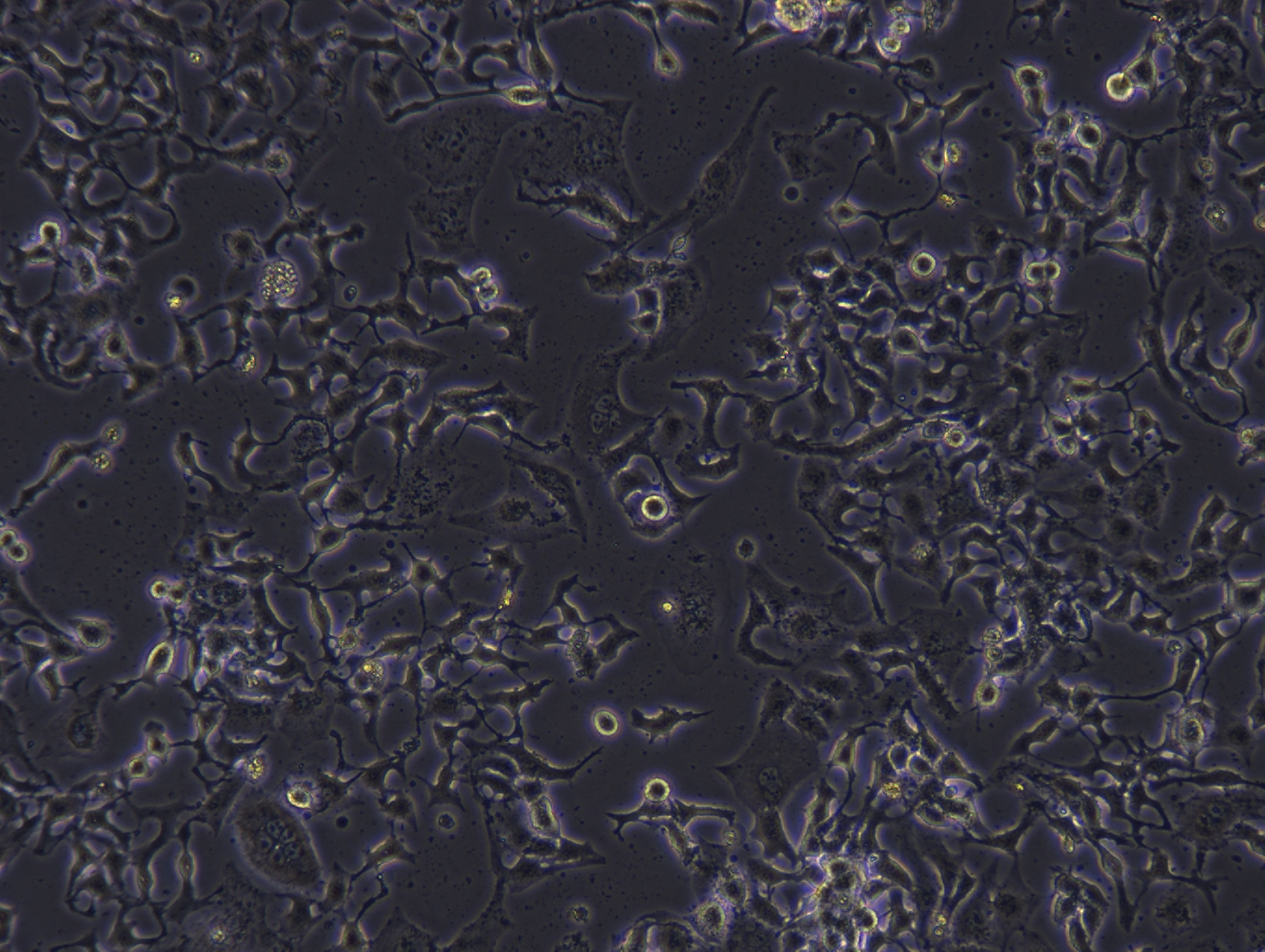
Representative picture of the morphology of MCF-7 cells before the development of Paclitaxel resistance.**

**
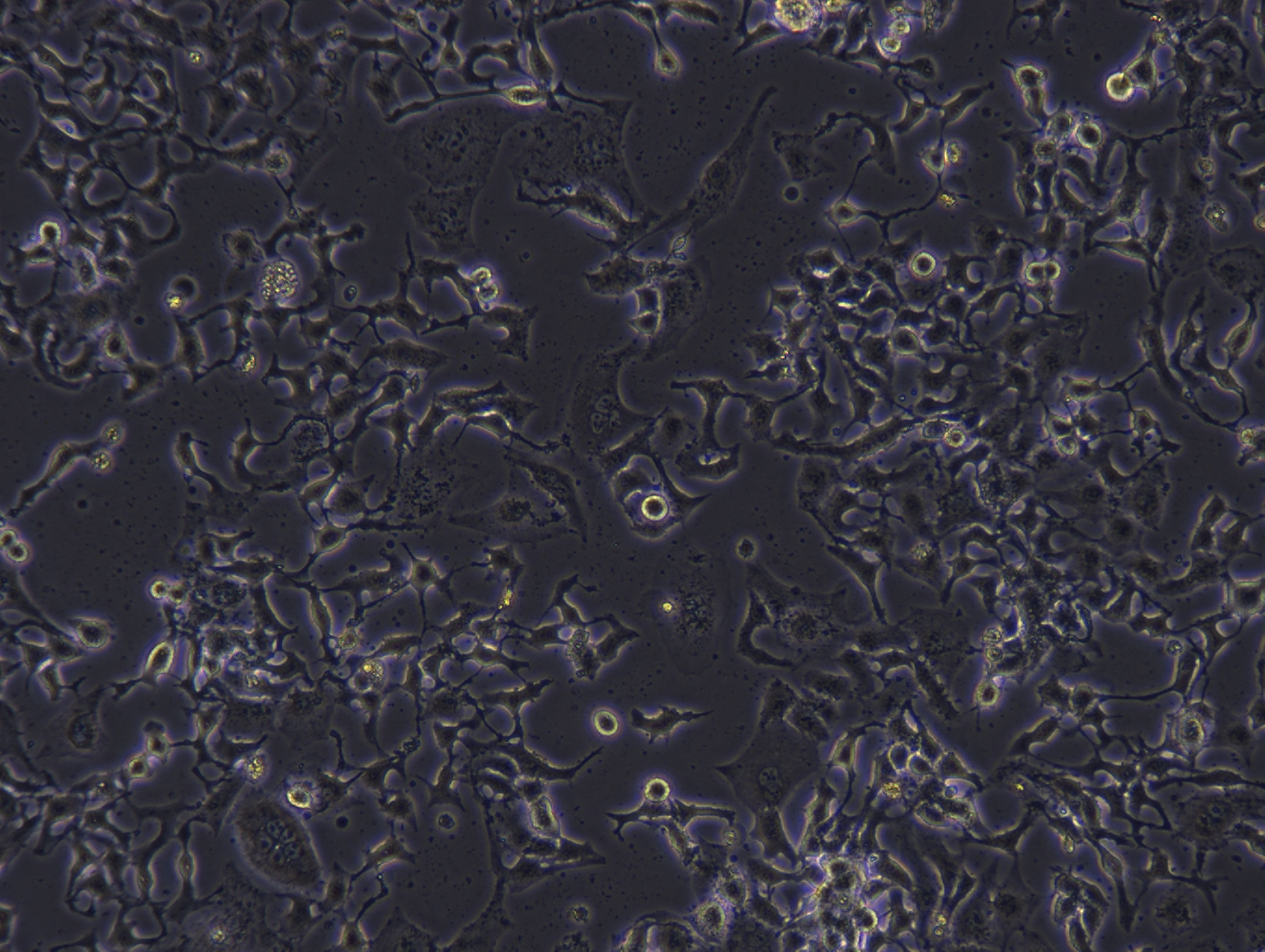
**

**Representative picture of the morphology of MCF-7 cells after the development of Paclitaxel resistance.**

**
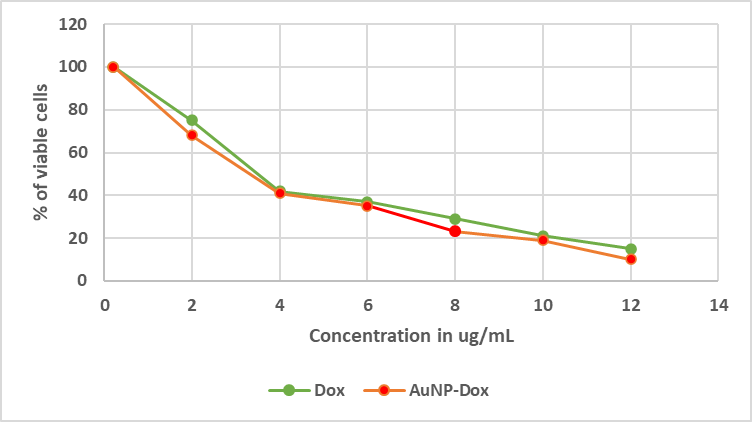

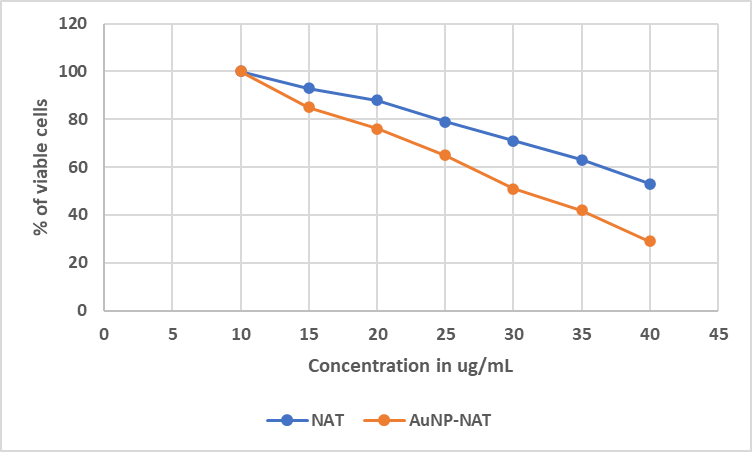
**

**Representative pictures of IC50 of the chosen drugs**

**
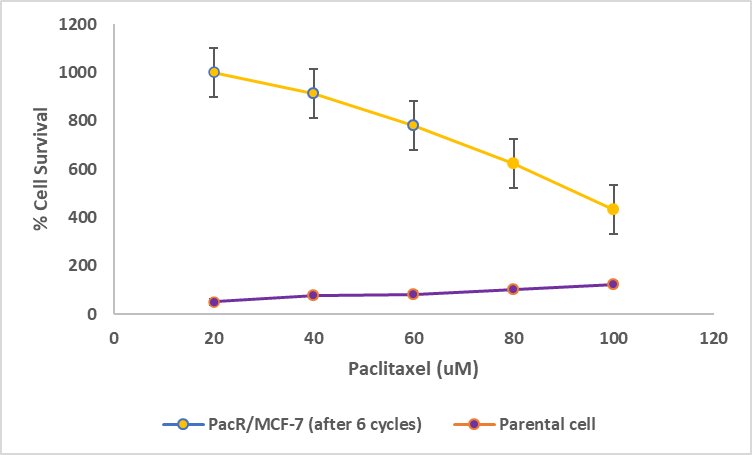
**

**Representative picture of increase in drug-resistance.**
